# The visual perception of aligned and crowded maxillary lateral incisors when smiling via eye tracking

**DOI:** 10.34172/joddd.2022.037

**Published:** 2022-12-30

**Authors:** Thiago Martins Meira, Gil Guilherme Gasparello, Oscar Mario Antelo, Jussimar Scheffer Castilhos, Mohamad Jamal Bark, Orlando Motohiro Tanaka

**Affiliations:** ^1^Department of Orthodontics, Bahia State University (UNEB), Guanambi, Brazil, School of Life Sciences, Pontifícia Universidade Católica do Paraná, Curitiba, Brazil; ^2^Department of Orthodontics, School of Life Sciences, Pontifícia Universidade Católica do Paraná, Curitiba, Brazil; ^3^Department of Orthodontics, Universidad Catolica Boliviana “San Pablo”, Santa Cruz de La Sierra, Bolivia, Pontifícia Universidade Católica do Paraná, School of Life Sciences, Curitiba, Brazil

**Keywords:** Alignment, Crowding, Esthetics, Eye tracking, Smile, Visual perception

## Abstract

**Background.:**

This study aimed to investigate whether the alignment of the teeth while smiling alters the visual perception by laypeople using eye tracking.

**Methods.:**

Facial images (two males and two females) were digitally edited to show a smile pattern with aligned teeth and one with crowded teeth. Sixty laypeople were selected to observe the images. The number of fixations, fixation duration, and time until the first fixation were recorded using an eye-tracking system. The results were qualitatively calculated with dot maps. Numerical data were analyzed using an independent Student’s *t *test.

**Results.:**

There were no significant differences in fixation duration and the number of fixations in the crowded smile, mainly that of the male. The fixation times for the teeth were significantly different when the participants viewed the male subjects with a crowded smile (*P*<0.05). Dot maps showed greater attention to the smile with crowded teeth in both genders.

**Conclusion.:**

The crowded maxillary incisor smile attracted more visual attention to males from laypeople.

## Introduction

The esthetics of the smile plays an important role in facial attractiveness.^1^ As the mouth is the center of communication in the face, the smile plays an important role in facial expression and appearance.^2^

An earlier study found attractiveness was equally important for men and women in most domains.^3^ An esthetically pleasing smile does not depend only on components such as tooth position, size, shape, and color; it also depends on the alignment display of the anterior teeth.

Smile esthetics has a significant impact on people’s quality of life.^4^ Dentistry can significantly modify and improve smile esthetics. If a balanced smile could decrease the visual perception of unesthetic characteristics, it might greatly benefit the patient.

With eye-tracking technology, the observer’s eye movements can be followed and recorded while viewing images. It is possible to quantify the direction of a person’s gaze, location, and duration.^5,6^

 Thus, this study aimed to investigate the effect of aligned and crowded maxillary incisors in a male and female smile. The null hypothesis was that there is no difference in laypeople’s visual perception regardless of tooth alignment.

## Methods

###  Samples

 This cross-sectional study comprised 60 participants (30 men and 30 women) with the following inclusion criteria: men or women aged 18–50, with the same racial pattern. Sample calculation was performed based on the heterogeneous population at 90% confidence level and 10% margin of error; the sample size was estimated at n = 60 subjects.

 All the participants signed a written informed consent form to participate in the study attesting they did not meet any of the items in the exclusion criteria, such as no neurological disabilities and no use of drugs that alter cognitive perceptions for 24 hours before the test.

###  Image design

 Facial frontal smiling photographs of one young male and one female without any significant facial discrepancy were obtained with a digital camera (Rebel XTI; Canon, Tokyo, Japan) in an illuminated studio environment against a white background. The selected photos were analyzed and standardized using the Photoshop program (Adobe Systems Inc, San Jose, California).

**Figure 1:**
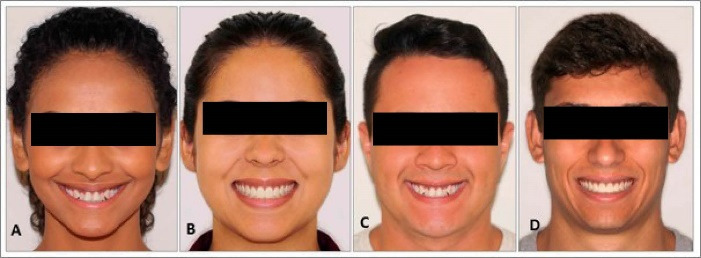
Images used in the study: Female with crowded teeth smile (A); Female with an aligned teeth smile (B); Male with a crowded teeth smile (C); Male with aligned teeth smile (D)

Initially, noise between layers, sharp contrast, and color were adjusted, followed by removing stains and spots and mirroring the image using the face of choice to obtain adequate symmetry. Aligned and leveled maxillary incisors were inserted into the two images (male and female), and another with crowded teeth with the maxillary lateral incisor positioned palatally was inserted in the other two images using Photoshop CS5 software (Figure 1).

 Those images were added to the Ogama software (Freie Universität, Berlin), and eye tracking was collected in conjunction with the Eye Tribe Tracker hardware (The Eye Tribe Aps, Copenhagen, Denmark).

###  Data collection

Previously, areas of interest (AOIs) comprising the mouth and the rest of the face were established on the models’ photographs to aid statistical comparison and allow the software to capture data, such as complete fixation time, time until first fixation, and several fixations, from the participants’ gaze (Figure 1). The AOIs were not visible in the photographs shown to the participants.

 The 60 observers were laypeople (no dentists or with no experience in clinical dentistry). Each participant viewed the images sitting down and positioned 60–90 cm from a high-definition Dell P2317 monitor (768 × 1366 pixels), oriented in an upright position to maintain the actual proportions of facial size, with the eye-tracking hardware positioned just below as recommended by the manufacturer. A 9-point calibration was then conducted by the software. The software calibration should be considered “perfect;” otherwise, the participant was excluded.

 The software displayed the processed eye movement data with dot maps and stored data on complete fixation time, time until the first fixation, and several fixations. The dot map showed the position of fixations on the image. The numerical data from each AOI was expressed in milliseconds.

Each previously-edited image was displayed on the screen for 3 seconds in random order performed by the website https://www.randomizer.org. To prevent the last focal point of an image from being the first on the following image, a blank green screen was displayed for 1 second between images. No details about the research were revealed until the experiment was completed.

###  Data analysis 

The results obtained from the eye-tracking software were tabulated in Microsoft Excel. Using univariate analysis, the eye-tracking variables (complete fixation time, time until the first fixation, and several fixations) were described by means and standard deviations. Independent samples Student’s *t* tests were used to calculate statistical significance between the AOI mouth in aligned and crowded teeth. A significance level of 5% was adopted. Data were analyzed using SPSS 25 (SPSS; SPSS Inc., Chicago, IL).

## Results

Descriptive statistics (means and standard deviations) of eye-tracking variables (complete fixation time, time until the first fixation, and several fixations) for different images regarding the mouth area of interest by gender are presented in Tables 1 and 2.

**Table 1:** Independent sample *t* test comparing AOI in male models

**Variable**	**Area**	**Aligned teeth**	**Crowded teeth**	***P*** ** value**
		**Mean (SD) (ms)**	**Mean (SD) (ms)**	
Complete fixation time	Mouth	66 (139.9)	1179 (383.3)	0.03^*^
Time until first fixation	Mouth	321 (716.7)	257.6 (545.2)	0.574
Number of fixation	Mouth	0.3 (0.6)	0.6 (0.7)	0.588

SD, standard deviation; ms, millisecond; AOI, areas of interest. * Statistical difference, *P* < 0.05

**Table 2:** Independent sample *t* test comparing AOI in female models

**Variable**	**Area**	**Aligned teeth**	**Crowded teeth**	***P*** ** value** ^*^
		**Mean (SD) (ms)**	**Mean (SD) (ms)**	
Complete fixation time	Mouth	144.2 (317.1)	143 (529.9)	0.983
Time until first fixation	Mouth	252 (575.6)	257.6 (545.2)	0.714
Number of fixation	Mouth	0.3 (0.6)	0.4 (0.7)	0.295

SD, standard deviation; ms, millisecond; AOI, areas of interest. * Statistical difference, *P* < 0.05

**Figure 2:**
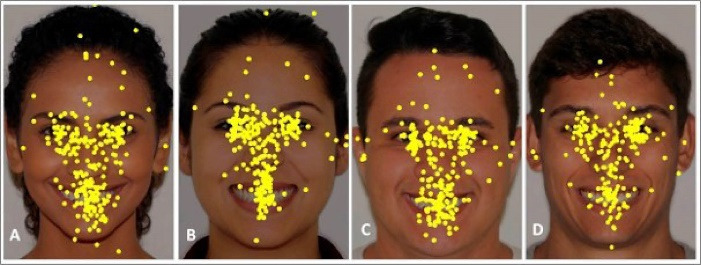
Dot maps: Female with a crowded teeth tic smile (A); Female with an aligned teeth smile (B); Male with a crowded teeth smile (C); Male with an aligned teeth smile(D)

Overall, dot maps showed that most attention was directed toward the eyes, nose, and mouth. The eyes are the most prominent facial feature, followed by the mouth (Figure 2). In the crowded teeth smile images, greater attention was directed toward the mouth (in the central area) compared to the aligned teeth smile images in both genders (Figure 2). The dot maps revealed a different pattern of gaze fixation between images. It was observed that on the aligned teeth smile images, the fixations were mostly in the central area of the smile. In the crowded teeth smile images, the points of gaze fixation were more spread around the mouth and chin (Figure 2).

Complete fixation time and the number of fixations on the other AOI were greater in the male crowded teeth smile than in the female, with no significant difference. Independent samples Student’s t-test showed a significant difference (*P* < 0.05) between the mean values of complete fixation time on the mouth for the male face (Table 1); the same was not observed for the female face (Table 2).

The number of fixations on the “other” AOI was higher in the crowded teeth smile images, with no significant difference, and this finding was more evident in the male face (Tables 1 and 2). The time until the first fixation on the mouth was lower for the crowded teeth smile images, which means that it called for more visual attention. No significant difference was found in the visualization patterns of the variables between the participants of both genders.

## Discussion

 This research investigated whether the presence of aligned or crowded maxillary incisors smile images impacted laypeople’s visual perception of the smile. Our findings showed that crowded teeth attracted more attention to the teeth, especially for male faces.

Our eye-tracking results indicated that crowded teeth drew more visual attention and were more noticeable than aligned ones. Another eye-tracking research showed that when dental attractiveness decreased, visual attention on the mouth increased, toward a level approaching that of the eyes.^7,8^

Dots map (Figure 2) showed that in images with aligned teeth, gaze fixation focused chiefly on the central area of the smile, especially on the central incisors. According to Machado,^9^ in the analysis of a balanced, beautiful smile, special attention is directed toward maxillary incisors. Our findings suggest that aligned teeth might attract attention to the incisors, especially the white esthetic teeth. On the contrary, in the presence of crowed teeth, gaze fixation points were spread more widely over the teeth (primarily), gingiva, and chin (Figure 2). Based on these findings, we hypothesize that crowded teeth disrupt the balance of facial esthetics, and other aspects, such as gingiva, may be more noticeable in the incisor area. Thus, our null hypothesis was rejected.

The tracking gaze parameters (complete fixation time, time until the first fixation, and several fixations) were different between male and female faces, consistent with Langlois et al.^3^ Differences in participants’ fixations on the teeth were more evident in the male face. A significant difference (*P* < 0.05) was found between the mean values of complete fixation time on the teeth for the male face (Table 1). The complete fixation time and the number of fixations on the gingiva also increased for the male face, although no significant difference was found (Table 1).

These differences between genders were interesting findings since most studies on visual perception in dentistry have not compared male and female faces and smiles.^5,7,10^ Previous studies have reported that in men, the smile was responsible for 49% and 69% of the perception of the attractiveness of aligned teeth by men and women, respectively.^11^ Our findings contradict these results since the change in smile esthetics did not have such an impact on the female face compared to the male face.

In dentistry, researchers in visual perception have become more interested in using laypeople as study participants^5,7,11^ because laypeople represent the view of patients, whose opinions are very important to clinicians developing a treatment plan in any specialty within dentistry.

Regarding the gender of the participants, no significant difference in the tracking variables was found in the visualization pattern of the teeth. However, female viewers directed significantly more visual attention to the eyes and chin than male participants. In contrast, male viewers directed more visual attention to the nose than female viewers. Another eye-tracking study revealed the same result.^7^

The duration of the display of each image in this study was 3 seconds which may reflect the first visual impression in an informal conversation or when people first meet. This choice was based on previous eye-tracking studies on facial and smile analysis.^5,7,12^ The literature has shown different display times of 2, 5, and 10 seconds for facial images.^8,10,13^ There is no consensus regarding whether a longer display time would change the pattern of visual attention and eye movements.

 In addition, a different degree of crowding of the upper central incisors may affect esthetics and smile perception. A comparison of these variables is suggested in future studies.

## Conclusion

 Based on the observations and results of this research, the following conclusions were drawn:

- Crowded maxillary incisors attracted more visual attention to the mouth, particularly for the male face.
- The eyes are the most prominent facial feature, followed by the nose and teeth.
- The gender of the viewer affected the duration of attention directed to the eyes, nose, and chin.

## Funding

 None.

## Ethics Approval

 Ethical approval for this study was obtained from the Ethics Committee of the Pontifícia Universidade Católica do Paraná under the reference number 3.729.413.

## Competing Interests

 The authors declare that they have no competing interests.
